# Atomistic Mechanisms and Temperature-Dependent Criteria of Trap Mutation in Vacancy–Helium Clusters in Tungsten

**DOI:** 10.3390/ma18153518

**Published:** 2025-07-27

**Authors:** Xiang-Shan Kong, Fang-Fang Ran, Chi Song

**Affiliations:** 1 State Key Laboratory of Advanced Equipment and Technology for Metal Forming, and Key Laboratory for Liquid-Solid Structural Evolution and Processing of Materials (Ministry of Education), Shandong University, Jinan 250061, China; 202400150214@mail.sdu.edu.cn; 2 College of Science, Jinling Institute of Technology, Nanjing 211169, China

**Keywords:** trap mutation, critical He/V ratio, temperature effect, tungsten, molecular dynamics simulations

## Abstract

Helium (He) accumulation in tungsten—widely used as a plasma-facing material in fusion reactors—can lead to clustering, trap mutation, and eventual formation of helium bubbles, critically impacting material performance. To clarify the atomic-scale mechanisms governing this process, we conducted systematic molecular statics and molecular dynamics simulations across a wide range of vacancy cluster sizes (n = 1–27) and temperatures (500–2000 K). We identified the onset of trap mutation through abrupt increases in tungsten atomic displacement. At 0 K, the critical helium-to-vacancy (He/V) ratio required to trigger mutation was found to scale inversely with cluster size, converging to ~5.6 for large clusters. At elevated temperatures, thermal activation lowered the mutation threshold and introduced a distinct He/V stability window. Below this window, clusters tend to dissociate; above it, trap mutation occurs with near certainty. This critical He/V ratio exhibits a linear dependence on temperature and can be described by a size- and temperature-dependent empirical relation. Our results provide a quantitative framework for predicting trap mutation behavior in tungsten, offering key input for multiscale models and informing the design of radiation-resistant materials for fusion applications.

## 1. Introduction

Tungsten is widely recognized as the leading plasma-facing material candidate for nuclear fusion reactors, owing to its exceptional physical and chemical properties, including a high melting point, excellent thermal conductivity, and strong resistance to sputtering erosion [1]. Despite these advantages, tungsten is subjected to severe degradation under the intense conditions of fusion environments, particularly due to high-flux helium ion irradiation [2,3,4]. Because helium has extremely low solubility and high mobility in tungsten, implanted He atoms readily become trapped at lattice defects such as vacancies or aggregate spontaneously to form He clusters. These clusters subsequently evolve into helium bubbles. The continued growth and rupture of such bubbles result in detrimental surface phenomena—blistering, pinhole formation, and the emergence of nanostructured fuzz—posing significant challenges to the operational stability and service lifetime of plasma-facing materials [2,3,4].

It has long been recognized that helium (He) atoms exhibit a strong affinity for vacancies in metals [5]. In the absence of accessible vacancies, helium atoms tend to aggregate to form mobile interstitial helium clusters [6,7,8]. The driving force behind this clustering is sufficiently strong that, at elevated helium concentrations, the accumulated elastic strain may be energetically relieved through the spontaneous ejection of one or more host atoms. This process results in the spontaneous displacement of host atoms from their lattice sites, giving rise to Frenkel pairs, namely vacancies (Vacs) and self-interstitial atoms (SIAs). The newly created vacancies can then trap the existing interstitial helium cluster, forming a vacancy–helium complex. The vacancy–helium complex continues to capture additional interstitial helium atoms. Upon reaching a critical size, the internal pressure again induces the emission of host atoms, resulting in the generation of further Frenkel pairs. This feedback mechanism enables the vacancy–helium complex to grow progressively and eventually evolve into a helium bubble. This sequence of helium-induced defect formation and complex evolution is commonly referred to as a self-trapping mutation (STM) process.

Atomistic simulations have become essential in materials science, not only corroborating experimental findings but also revealing the underlying mechanisms that drive numerous physical processes, many of which are beyond the scope of traditional experimental methods. So far, comprehensive computational studies have extensively examined the thermodynamic and kinetic behaviors involved in the STM process, including the dissolution, migration, and self-clustering of interstitial helium and its aggregation at vacancies in tungsten [6,7,8,9,10,11,12,13,14,15,16,17,18]. Theoretical investigations revealed that interstitial helium atoms preferentially occupy tetrahedral interstitial sites in tungsten, exhibiting exceptionally low migration barriers (0.06 eV) [7,8]. Strong attractive interactions exist between interstitial helium atoms, with binding energies reaching up to 1.07 eV for helium dimers—indicating robust clustering tendencies [9,10,11]. As cluster size increases, the affinity of helium clusters toward isolated neighboring helium interstitials is enhanced, reaching up to approximately 2.5 eV for larger clusters [9,10,11]. Thermodynamic analyses suggest that clusters exceeding six helium atoms trigger trap mutation events, characterized by the emission of an SIA and the formation of vacancy–helium (${Vac}_{n}{He}_{m}$) complexes [9,10,11].

Concurrently, ${Vac}_{n}{He}_{m}$ complexes have also been extensively investigated through atomistic simulations, with a primary focus on their structural stability and energetic properties [9,10,11,12,13,14,15,16,17,18,19,20,21,22,23,24,25,26]. These studies have consistently shown that helium atoms in ${Vac}_{n}{He}_{m}$ complexes tend to occupy central positions within vacancy clusters, and as the He/V ratio increases, helium atoms form compact clusters [9,10,11,12,13,14,15,16,17,18]. In addition, the minimum atomic distances between He–He and He–W decrease sharply with increasing helium content, stabilizing at approximately 1.53 Å and 1.83 Å, respectively [18]. Simultaneously, the binding energy of interstitial helium to ${Vac}_{n}{He}_{m}$ clusters drops rapidly from values as high as ~6 eV at low He/V ratios and plateaus near ~3 eV when the ratio approaches 5 to 6 [18,19]. Building upon ab initio calculations, Jiang et al. proposed a unified theoretical framework to describe the energetics of noble gas bubbles in body-centered cubic (bcc) transition metals [26]. More recently, Song and co-workers refined this model for tungsten by incorporating high-resolution structural and energetic insights obtained from first-principles simulations [18]. Thermodynamic analysis based on the updated model indicates that as Vac–He complex sizes increase, the He/V ratio for thermodynamically stable configurations generally ranges between 1.5 and 3, while the threshold He/V ratio for initiating trap mutation lies between 5 and 6.5 [18]. However, conventional thermodynamic assessments are typically conducted at 0 K, which inherently neglects the critical role of temperature in governing the trap mutation behavior of ${Vac}_{n}{He}_{m}$ complexes.

Thus, despite substantial progress, a critical knowledge gap remains: the lack of a precise, temperature-dependent characterization of the cluster size or He/V ratio required to initiate trap mutation in ${Vac}_{n}{He}_{m}$ complexes. This limitation hampers our ability to accurately simulate helium bubble nucleation and growth in tungsten, as trap mutation is a key mechanism driving microstructural evolution under irradiation. Addressing this gap is essential for developing predictive multiscale models capable of capturing the intricate coupling among helium aggregation, vacancy dynamics, and defect evolution in fusion-relevant tungsten environments.

In this study, we employed a combined approach of molecular statics (MS) and molecular dynamics (MD) simulations to systematically elucidate the trap mutation behavior of ${Vac}_{n}{He}_{m}$ clusters in tungsten. By constructing clusters with vacancy numbers ranging from 1 to 27 and simulating across a wide temperature spectrum, we quantitatively mapped the interplay between the cluster size and thermal activation on the onset of trap mutation. Our findings reveal that the critical He/V ratio required to trigger trap mutation decreases monotonically with increasing vacancy number, converging to approximately 5.6 at 0 K. More notably, temperature exerts a pronounced linear effect: as the temperature increases, the threshold He/V ratio for mutation decreases accordingly, indicating that thermal energy significantly facilitates the emission of SIAs. These insights provide a fundamental basis for understanding helium bubble nucleation and growth in fusion-grade tungsten, highlighting the critical role of thermally activated processes in the microstructural evolution under irradiation. Furthermore, the mutation thresholds identified here offer essential input parameters for coarse-grained simulations such as kinetic Monte Carlo, enabling more accurate predictions of helium bubble dynamics and aiding in the rational design of radiation-tolerant, tungsten-based materials for next-generation fusion reactors.

## 2. Computational Methods

**Figure 1:**
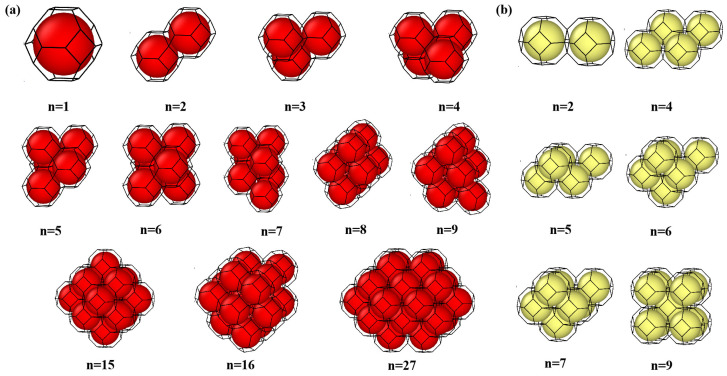
(**a**) Atomistic structures of stable vacancy clusters (n = 1–9, 15, 16, and 27), determined by minimizing Wigner–Seitz area, where red spheres indicate constituting vacancies and black lines show edges of Wigner–Seitz cells on the vacancy cluster surface. (**b**) Atomistic structures of meta-stable vacancy clusters (n = 2, 4–7, and 9).

To unravel the temperature- and size-dependent behavior of ${Vac}_{n}{He}_{m}$ complexes in tungsten, we performed a comprehensive suite of MS and MD simulations using the LAMMPS code (Stable Release 29 August 2024) [27]. The W-He potential developed by Juslin et al. [28] was used in this work, which has previously demonstrated reliable accuracy in reproducing helium clustering behavior in tungsten vacancies [17]. All simulations were carried out within a 10 × 10 × 10 body-centered cubic (bcc) tungsten supercell containing 2000 atomic sites. To ensure the adequacy of this cell size, convergence tests were performed using larger supercells. These tests revealed no significant deviations in results, confirming that the selected simulation cell reliably captures the relevant physical behavior. Vacancy clusters containing n vacancies were systematically constructed following the Wigner–Seitz surface minimization protocol proposed by Hou et al. [29], encompassing cluster sizes of n = 1–9, 15, 16, and 27. This construction method ensures highly stable configurations by minimizing the surface area of the void. Figure 1a illustrates the representative configurations corresponding to the minimum Wigner–Seitz area for selected cluster sizes. In addition to these energetically favored geometries, several metastable configurations were also considered to capture the structural diversity and to assess their influence on helium trapping and evolution behavior. To construct ${Vac}_{n}{He}_{m}$ clusters, m helium atoms were randomly introduced into each ${Vac}_{n}$ cluster. For each configuration, 100 unique atomic arrangements were generated via randomized seeding and then relaxed at 0 K using conjugate gradient energy minimization to identify the global minimum-energy structure. These structures served as the initial configurations for further analysis.

To investigate the temperature dependence of trap mutation in ${Vac}_{n}{He}_{m}$ clusters, four representative temperatures (500, 1000, 1500, and 2000 K) were selected. This temperature range was chosen based on the high-temperature service conditions of tungsten as a plasma-facing material (typically ~1000 K), as well as helium thermal desorption spectroscopy (TDS) experiments, which report desorption peaks distributed between 500 and 2000 K, corresponding to helium release from clusters of varying sizes.

The simulation consisted of two parts: (i) At 0 K, molecular statics (MS) simulations were performed using the conjugate gradient algorithm to obtain the energy-minimized atomic configurations. The energy and force convergence criteria were set to 1 × 10^−10^ eV and 1 × 10^−10^ eV/Å, respectively. (ii) At finite temperatures, canonical (NVT) ensemble molecular dynamics (MD) simulations were carried out to capture the temperature-induced behavior of the clusters. The use of NVT ensemble under similar conditions is consistent with prior atomistic studies on helium behavior in tungsten (e.g., Refs. [17,18]). Each simulation was performed with a time step of 0.001 ps and a total duration of 1 ns. Periodic boundary conditions were used throughout this study. This simulation framework enabled a rigorous investigation of helium behavior under realistic thermal and structural conditions, laying the groundwork for deeper insights into bubble formation in tungsten under irradiation.

## 3. Results and Discussion

### 3.1. Atomic Displacement as a Signature of Trap Mutation in Helium-Loaded Vacancy Clusters

As helium atoms progressively accumulate within the vacancy clusters, the internal pressure increases, inducing growing lattice distortion in the surrounding tungsten atoms. Once a critical number of helium atoms is reached, this buildup of stress becomes sufficient to eject the SIA, thereby relieving the excess pressure. To detect the onset of such trap mutation events, we systematically tracked the atomic distortion in the tungsten lattice as a function of He loading. For each ${Vac}_{n}{He}_{m}$ configuration, we analyzed the lowest-energy structure obtained from MS calculations and computed the maximum displacement of W atoms relative to their reference positions—defined as the relaxed structure of ${Vac}_{n}$ without helium. The atomic displacement $d_{W}$ was calculated using the following equation:

(1) $$d_{W}=\sqrt{{\left(x-x_{ref}\right)}^{2}+{\left(y-y_{ref}\right)}^{2}+{\left(z-z_{ref}\right)}^{2}},$$

where ($x_{ref}$, $x_{ref}$, $x_{ref}$) are the coordinates in the reference state.

**Figure 2:**
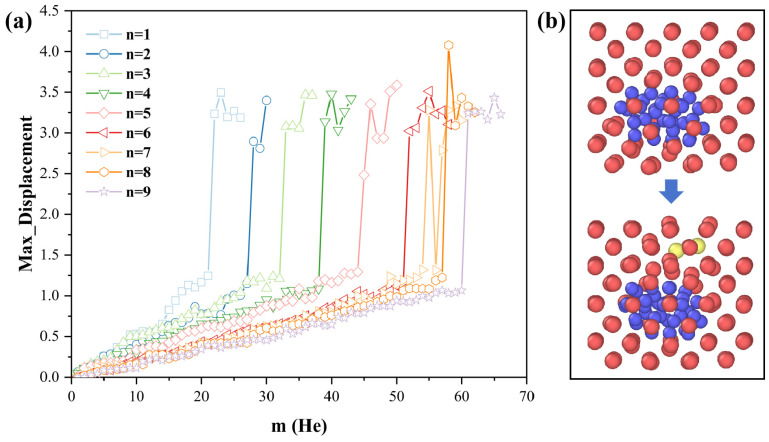
Identification of trap mutation via atomic displacement in ${Vac}_{n}{He}_{m}$ clusters at 0 K. (**a**) Maximum tungsten atomic displacement as a function of helium atom number for different vacancy cluster sizes (n = 1–9). A near-linear increase in displacement is observed with increasing He loading, followed by a sudden jump indicative of trap mutation, where displacement exceeds the W–W nearest-neighbor distance (2.74 Å). (**b**) Atomic configurations of a representative ${Vac}_{n}{He}_{m}$ cluster before and after trap mutation (**top**: ${Vac}_{4}{He}_{38}$ cluster; **bottom**: ${Vac}_{4}{He}_{39}$ cluster). Only atoms in the vicinity of the clusters are displayed. Blue spheres represent He atoms, red spheres denote lattice W atoms, and yellow spheres indicate displaced SIAs. The structural transition reveals the formation of SIAs near the vacancy cluster and additional vacancies inside the cluster, confirming the occurrence of trap mutation.

As shown in Figure 2a, across all studied clusters, the maximum W atomic displacement increases nearly linearly with the number of helium atoms, reflecting the gradual lattice deformation induced by the internal gas pressure. However, beyond a specific He concentration, this displacement exhibits a sudden jump—from ~1 Å to approximately 3 Å—exceeding the nearest-neighbor W–W bond length in pristine bcc tungsten (2.74 Å). This abrupt increase signals a large-scale atomic shift, suggestive of atomic extrusion from the lattice. Here, for vacancy cluster sizes ranging from n = 1 to 9, our simulations reveal that the critical numbers of helium atoms required to trigger trap mutation are 22, 28, 33, 39, 45, 52, 55, 58, and 61, respectively. The Wigner–Seitz defect analysis confirms that, at this point, self-interstitial atoms emerge near the cavity, while the number of vacancies within the vacancy clusters increases, which are hallmarks of the trap mutation mechanism. It is noteworthy that, as shown in Figure 2a, the maximum displacement values for most ${Vac}_{n}{He}_{m}$ clusters range around 3 Å, which corresponds to the displacement of a W atom to a first-nearest-neighbor (1NN) site during trap mutation. However, in the case of n = 8, a larger displacement (~4 Å) is observed. Further structural analysis reveals that the displaced W atom in this case is pushed to a position near the second-nearest-neighbor (2NN) site, rather than 1NN, thus resulting in the observed deviation. Figure 2b illustrates the structural transition of a representative ${Vac}_{n}{He}_{m}$ cluster before and after trap mutation, clearly highlighting the local atomic rearrangements and SIA formation. Therefore, we designate the abrupt surge in the maximum W atomic displacement as a definitive signature of trap mutation in helium-loaded vacancy clusters.

### 3.2. Atomistic Mechanisms of Trap Mutation at 0 K: Critical Thresholds and Structural Evolution

Building upon these observations, we extended our analysis across a broader range of vacancy cluster sizes—including n = 15, 16, and 27—to establish a generalizable relationship between vacancy cluster size and mutation propensity at 0 K. By normalizing the critical number of helium atoms with respect to the number of vacancies, we obtained a set of critical He/V ratios that quantitatively capture the size-dependent mutation threshold across clusters of varying complexity.

**Figure 3:**
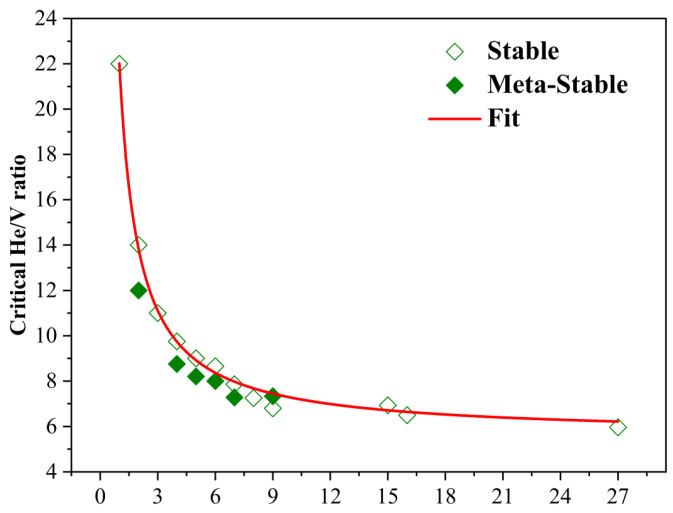
Critical He/V ratios for trap mutation in ${Vac}_{n}{He}_{m}$ clusters at 0 K. Variation of the critical He/V ratio as a function of vacancy cluster size (n = 1–9, 15, 16, 27), obtained from MS simulations by identifying the onset of trap mutation via a sudden jump in the maximum tungsten atomic displacement. Both the most stable (hollow squares) and selected metastable (solid squares) vacancy cluster configurations are considered. The red curve represents the empirical fitting using Equation (2).

As illustrated in Figure 3, the critical He/V ratio exhibits a pronounced inverse relationship with the cluster size: it decreases rapidly for small n, then gradually converges toward an asymptotic limit. This trend suggests a diminishing marginal effect of additional vacancies on the pressure build-up required to trigger mutation. This empirical correlation is well described by a simple rational function:

(2) $$R_{c}=A+\frac{B}{n},$$

where $R_{c}$ denotes the critical He/V ratio, and n is the vacancy number. Fitting yields A=5.48 and B=16.52, indicating that the mutation threshold asymptotically approaches 5.6 for large clusters—consistent with previous thermodynamic predictions based on the binding energies between vacancy–helium clusters and individual helium atoms or vacancies [18]. Notably, the deviation between our results and thermodynamic estimates is most prominent in small clusters (e.g., n ≤ 6), where the critical He/V ratios are substantially higher than 10 [18].

To assess the robustness of this critical ratio, we further evaluated the metastable vacancy cluster geometries (as shown in Figure 1b) for selected sizes (n = 2, 4, 5, 6, 7, 9). The corresponding mutation thresholds (also plotted in Figure 3) showed some structural dependence, but the overall size trend and critical He/V scaling remained largely intact. This indicates that while local vacancy geometry may slightly modulate the stress distribution and atomic mobility, the He/V-based mutation threshold provides a generally reliable descriptor for trap mutation behavior across realistic defect configurations.

**Figure 4:**
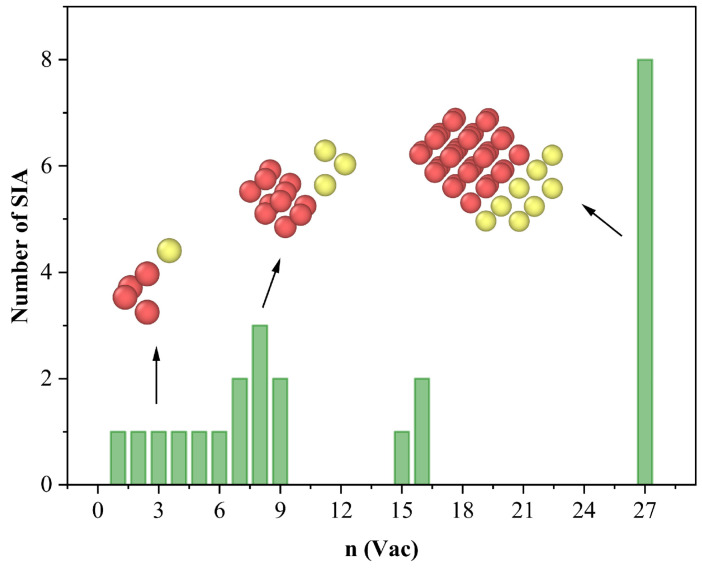
Size-dependent SIA extrusion during trap mutation of ${Vac}_{n}{He}_{m}$ clusters. The number of SIAs extruded from ${Vac}_{n}{He}_{m}$ clusters at their respective trap mutation thresholds is plotted as a function of vacancy cluster size n. Insets illustrate representative SIA extrusion configurations (red and yellow spheres indicate the positions of vacancies and SIAs after trap mutation, respectively) and highlight differences between different vacancy–helium clusters.

To further elucidate the structural consequences of trap mutation, we evaluated the number of SIAs expelled from the ${Vac}_{n}{He}_{m}$ clusters at their respective mutation thresholds. These results, summarized in Figure 4, reveal a clear size-dependent behavior. For small vacancy clusters (n ≤ 6), the trap mutation process typically ejects SIAs in a sequential, one-by-one manner. For instance, in the case of a three-vacancy cluster, a single tungsten atom is displaced, transforming the cluster into a tetrahedral four-vacancy configuration—a geometry previously identified as energetically favorable. In contrast, larger clusters (n ≥ 7) often experience more abrupt structural transitions, with multiple SIAs being expelled simultaneously during trap mutation. For example, in the n = 8 case, three SIAs are ejected in a single event. This behavior also seems to correlate with the intrinsic symmetry and stability of the vacancy cluster. Clusters with highly symmetric geometries, such as the n = 15 rhombic dodecahedron, tend to undergo minimal atomic rearrangement, ejecting only a single SIA. Conversely, less-ordered configurations—such as the irregular polyhedral structure observed in the n = 27 cluster—are associated with the simultaneous extrusion of up to eight SIAs. Due to the displaced W atoms forming SIAs, the vacancy count of the ${Vac}_{n}{He}_{m}$ cluster increases. For example, a ${Vac}_{8}{He}_{m}$ cluster may evolve into a structure containing ~11 vacancies post-mutation. This transformation releases internal pressure caused by helium, reduces system energy, and yields a more stable configuration. The generated SIAs remain around the cluster due to strong binding. Under certain conditions, these SIAs may reorganize into dislocation loops and detach from the cluster—a process known as dislocation loop punching—which plays a critical role in helium bubble nucleation and growth [9].

Interestingly, the trap mutation process drives the ${Vac}_{n}{He}_{m}$ clusters to evolve along a well-defined structural pathway that reflects energetic optimization. Starting from a monovacancy, the trap mutation process sequentially builds up vacancy clusters that closely match the most stable configurations identified in prior studies. For example, a monovacancy evolves into a <111>-aligned divacancy, then into a triangular trivacancy, followed by a tetrahedral four-vacancy cluster (as illustrated in Figure 1). This observation underscores the thermodynamic preference for specific void geometries during trap mutation, suggesting that helium-loaded clusters naturally evolve toward structurally optimized configurations. Therefore, in all subsequent analyses, our simulations will focus on these most stable geometries as representative cases.

### 3.3. Thermally Activated Trap Mutation and Stability Thresholds of ${Vac}_{n}{He}_{m}$ Clusters

**Figure 5:**
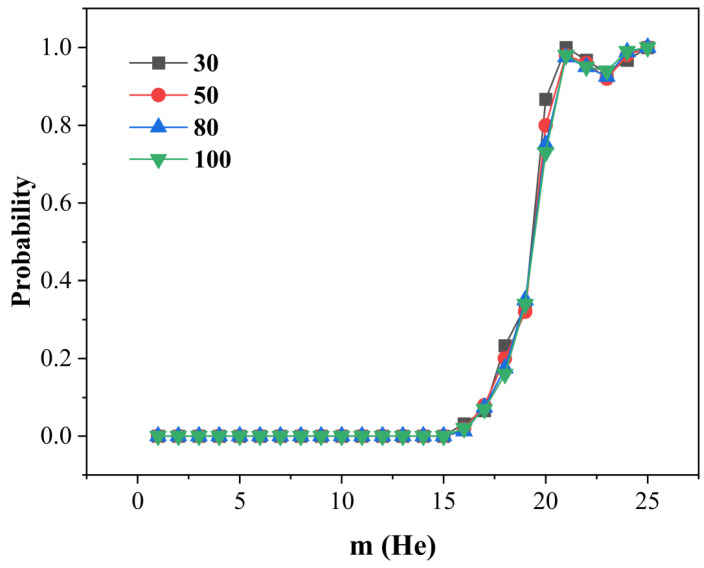
Convergence of trap mutation probability with simulation count for ${Vac}_{1}{He}_{m}$ clusters. At 300 K, the trap mutation probability $P_{t}$ was evaluated for ${Vac}_{1}{He}_{m}$ clusters using N = 30, 50, 80, and 100 independent MD simulations.

Temperature plays a pivotal role in governing the trap mutation behavior of helium-vacancy clusters. We performed temperature-controlled MD simulations to systematically evaluate its impact. For each ${Vac}_{n}{He}_{m}$ configuration, we carried out N independent MD runs at a given temperature, each lasting 1 ns. During these simulations, trap mutation events were actively monitored in real-time by detecting large displacements of tungsten atoms, which serve as a hallmark of SIA emission. We defined the probability of trap mutation, $P_{t}$, as the ratio of simulations in which the mutation occurred ($N_{t}$) to the total number of runs: $P_{t}=N_{t}/N$. To ensure statistical reliability, we first evaluated the convergence behavior of $P_{t}$ using the representative ${Vac}_{1}{He}_{m}$ system. As shown in Figure 5, $P_{t}$ becomes statistically stable once the number of simulations exceeds 30. Therefore, in all subsequent MD simulations across different cluster sizes and temperatures, we adopted N=30 to balance computational efficiency with statistical accuracy.

**Figure 6:**
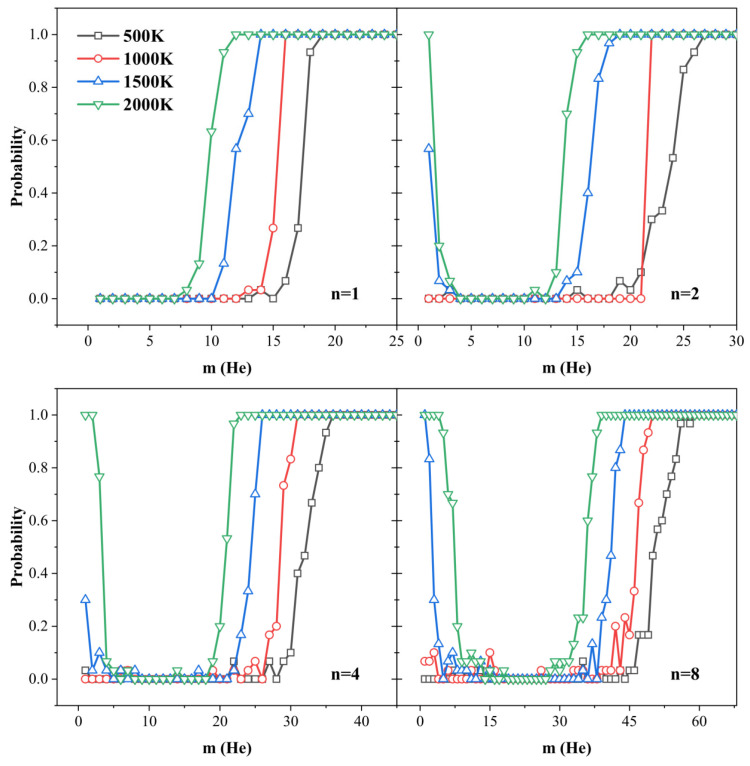
Trap mutation probability for ${Vac}_{n}{He}_{m}$ clusters with n = 1, 2, 4, and 8 at 500, 1000, 1500, and 2000 K.

**Figure 7:**
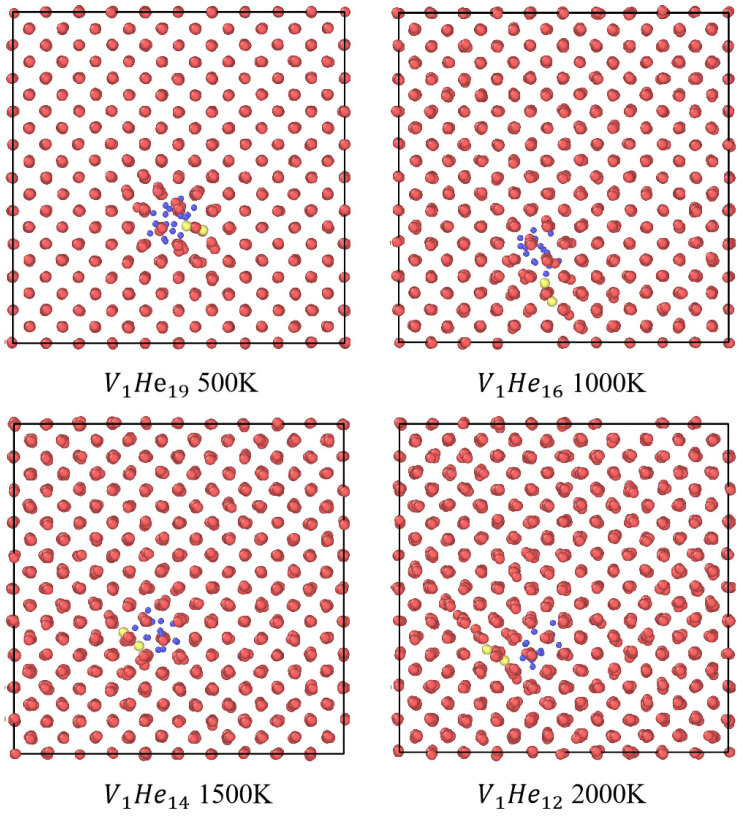
Atomic snapshots of a monovacancy system with helium before and after trap mutation at 500 K ($V_{1}{He}_{19}$), 1000 K ($V_{1}{He}_{16}$), 1500 K ($V_{1}{He}_{14}$) and 2000 K ($V_{1}{He}_{12}$). Blue spheres represent helium atoms, red spheres denote lattice W atoms, and yellow spheres indicate displaced SIAs.

To further elucidate the influence of temperature on the trap mutation behavior, we carried out MD simulations at four representative temperatures: 500 K, 1000 K, 1500 K, and 2000 K. Figure 6 presents the temperature-dependent evolution of the trap mutation probability $P_{t}$ as a function of the number of helium atoms within each cluster. Here, for clarity, four representative ${Vac}_{n}{He}_{m}$ clusters with n = 1, 2, 4, and 8 were selected. To visually support the findings on the temperature-dependent trap mutation behavior, we present atomic configurations before and after trap mutation at selected temperatures for the monovacancy case (see Figure 7). These snapshots provide microscopic insight into the structural transformation induced by He insertion. It is evident that as the temperature increases from 500 K to 2000 K, the number of helium atoms required to trigger trap mutation gradually decreases. This trend reflects the enhanced atomic mobility and reduced energy barrier for structural transition at elevated temperatures.

Due to the extremely small size of helium clusters during the early stages of He bubble nucleation and growth, experimental detection of the exact number of helium atoms within a vacancy cluster is currently infeasible. Consequently, determining the critical helium content required for trap mutation remains experimentally inaccessible. This limitation underscores the importance of atomistic simulations, which can offer valuable insights into the underlying mechanisms governing such thermally activated processes.

At lower temperatures (500 K and 1000 K), all clusters exhibit a sharp, step-like transition in the mutation behavior. Initially, $P_{t}$ remains nearly zero when the helium content is below a critical threshold, indicating negligible trap mutation activity. As the number of helium atoms surpasses this threshold, $P_{t}$ rises abruptly and saturates at 1, suggesting that once a critical internal pressure is reached, trap mutation occurs with near certainty. However, a distinct response emerges at higher temperatures (1500 K and 2000 K). While the same saturation behavior is observed at high-helium loadings, a significant deviation occurs in the low-helium regime—particularly for clusters ${Vac}_{n}{He}_{m}$ with n > 1. In this regime, non-negligible trap mutation probabilities are recorded, even when only one or two helium atoms are present, sometimes reaching values close to 1. This behavior is not a statistical artifact but rather a consequence of an alternative mechanism: thermally driven dissociation of the vacancy clusters.

**Figure 8:**
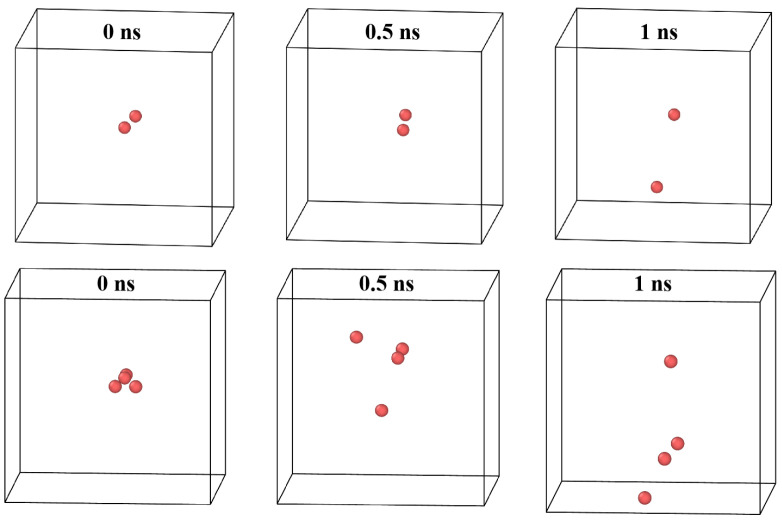
Visualized vacancy coordinates from MD simulations in ${Vac}_{2}{He}_{1}$ (**top**) and ${Vac}_{4}{He}_{1}$ (**bottom**) clusters at 1500 K. Structures shown at 0, 0.5, and 1 ns.

Importantly, $P_{t}$ is defined based on the detection of large displacements of tungsten atoms, which—at high temperatures—can also result from the breakup of weakly bound vacancy clusters. This is especially relevant for small clusters with low or even negative binding energies (e.g., di-vacancies), which are prone to thermal decomposition under high thermal agitation. To validate this hypothesis, we examined the behavior of ${Vac}_{2}{He}_{1}$ and ${Vac}_{4}{He}_{1}$ clusters at 1500 K and visualized the spatial distribution of vacancies over time (Figure 8). In both cases, the original cluster dissociated into two isolated vacancies. The helium atom remained strongly bound to one of the vacancies, while the other migrated over long distances—resulting in substantial atomic displacements that mimic the signature of trap mutation. This observation underscores a critical insight: small vacancy clusters become thermodynamically unstable at elevated temperatures and tend to dissociate unless they are stabilized by helium. As the helium content increases, this dissociation behavior is suppressed, and $P_{t}$ returns to zero in the subcritical helium regime. These results highlight the stabilizing effect of helium on the vacancy clusters.

The above findings reveal that for all ${Vac}_{n}{He}_{m}$ clusters with n > 1, there exists a well-defined range of He/V ratios within which the clusters remain structurally stable at elevated temperatures. Below this stability window, the limited number of helium atoms offers insufficient binding strength to suppress thermal vacancy dissociation, leading to the fragmentation of the cluster and a reduction in its size. Conversely, when the He/V ratio exceeds the upper bound of this window, the internal pressure increases to a critical level, triggering trap mutation and the emission of SIAs, thereby increasing the cluster size.

**Figure 9:**
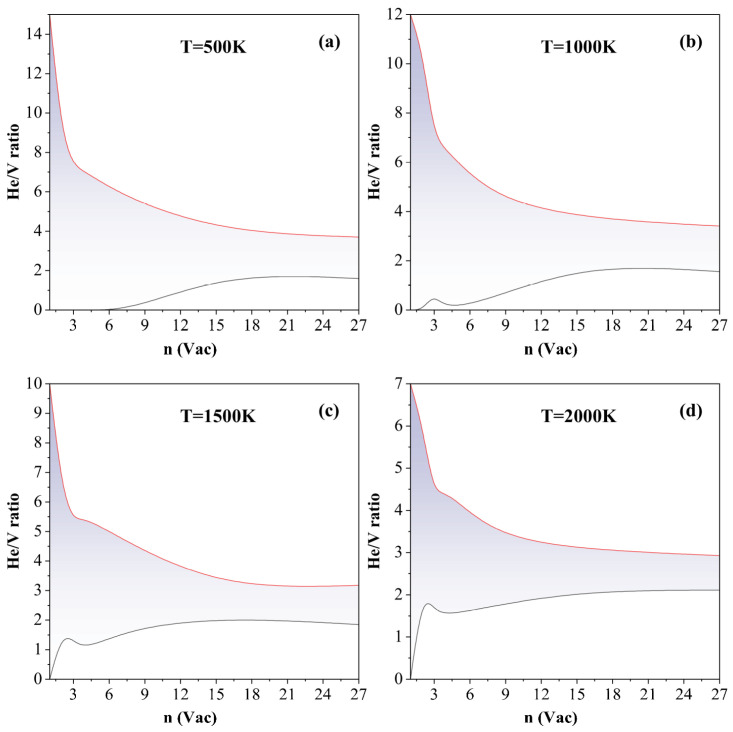
Size and temperature dependence of the stable He/V ratio window in helium–vacancy clusters. The shaded regions represent the He/V ratio intervals in which the trap mutation probability $P_{t}$ remains approximately zero, indicating structural stability of the ${Vac}_{n}{He}_{m}$ clusters. Data are shown for four representative temperatures: (**a**) 500 K, (**b**) 1000 K, (**c**) 1500 K, and (**d**) 2000 K.

We define this stability window operationally as the region where the trap mutation probability $P_{t}$ remains approximately zero—corresponding to the “valley” region in the probability profiles shown in Figure 6. Figure 9 summarizes the evolution of this stable He/V range with the vacancy cluster size at various temperatures. Across all conditions, a consistent trend emerges: the width of the stable He/V window decreases rapidly with increasing cluster size and eventually converges to a narrow range. At 1000 K, the stability window narrows to between approximately 1.6 and 3.4 for large clusters. With further increases in temperature, the window becomes even more constrained: at 1500 K and 2000 K, the stability ranges tighten to 1.9–3.2 and 2.1–2.9, respectively. These narrow intervals represent the helium concentrations at which most helium bubbles are likely to persist without undergoing structural transformation or disintegration.

Remarkably, our findings are in excellent agreement with previous long-timescale MD simulations by Hammond et al., who studied the helium bubble evolution in tungsten subjected to low-energy helium ion implantation [30]. Their results indicated that for helium bubbles located deep beneath the surface and containing more than 50 vacancies, the He/V ratio remains consistently within the narrow range of 2 to 3 throughout the simulation period. This consistency across the methodologies underscores the universality of the He/V stability window.

**Figure 10:**
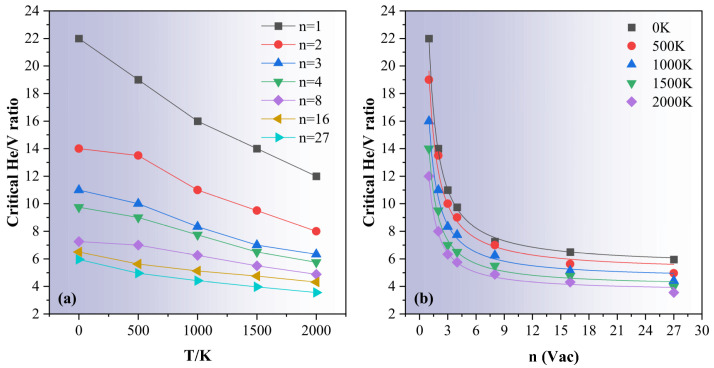
Temperature and size dependence of the critical He/V ratio for trap mutation. (**a**) Evolution of the critical He/V ratio with temperature for vacancy–helium clusters of different sizes. (**b**) Size dependence of the critical He/V ratio at various temperatures (500 K, 1000 K, 1500 K, and 2000 K). The solid lines represent fits using Equation (2), with temperature-dependent parameters $A=5.48-9.56\times 10^{-4}T$ and $B=16.52-4.18\times 10^{-3}T$.

To extend the trap mutation criterion established at 0 K into finite-temperature regimes, we defined the critical condition for trap mutation at a given temperature as the point at which the trap mutation probability $P_{t}$ first reaches unity as a function of helium loading. As shown in Figure 6, the critical number of helium atoms required to trigger trap mutation systematically decreases with increasing temperature for any given vacancy cluster size. This indicates that thermal energy facilitates the structural instability associated with helium over-pressurization, thereby lowering the mutation threshold. To further quantify this temperature dependence, Figure 10a presents the variation of the critical He/V ratio as a function of temperature for clusters of various sizes. Across all configurations examined, the critical He/V ratio decreases linearly with increasing temperature. This linear trend strongly suggests that thermal activation enhances the propensity for trap mutation by lowering the mechanical stress threshold required to trigger atomic ejection. Conversely, at any fixed temperature, the critical He/V ratio also exhibits a systematic dependence on cluster size. As illustrated in Figure 10b, the critical ratio declines rapidly with increasing vacancy number and approaches a saturation value, which is an asymptotic limit that itself decreases with increasing temperature. This observation is consistent with our findings at 0 K but now is extended to thermally activated regimes. We fitted the simulation data using Equation (2), wherein the fitting parameters A and B are explicitly temperature-dependent. We found excellent agreement across all temperatures using linear forms: $A=5.48-9.56\times 10^{-4}T$ and $B=16.52-4.18\times 10^{-3}T$. This formulation not only captures the full range of behaviors observed in the simulations but also provides predictive insight: for large helium bubbles, the asymptotic value A represents the limiting He/V ratio necessary to induce trap mutation at a given temperature. This metric is of particular importance in coarse-grained models of helium bubble evolution under irradiation, enabling the implementation of temperature-dependent mutation criteria grounded in atomistic physics.

## 4. Conclusions

In this study, we systematically investigated the atomic-scale mechanisms governing trap mutation in helium-loaded vacancy clusters in tungsten across a range of cluster sizes and temperatures. Our findings offer a unified, quantitative framework for understanding the onset and evolution of trap mutation phenomena under irradiation-relevant conditions.

At 0 K, we identified an abrupt increase in atomic displacement as a reliable structural signature of trap mutation. The critical condition for mutation was found to be strongly size-dependent, with the critical He/V ratio decreasing inversely with cluster size. This relationship was well captured by a rational empirical model, with an asymptotic limit of approximately 5.6, which is consistent with thermodynamic expectations. Structural analyses revealed that trap mutation drives vacancy clusters toward energetically favorable geometries through sequential SIA emission, with the number and mode of SIA ejections closely tied to the cluster’s symmetry and size.

At finite temperatures, the thermal fluctuations introduce additional dynamics. We found that vacancy clusters exhibit a well-defined He/V stability window. Below this range, clusters are prone to thermally activated dissociation; above this range, the mutation is triggered by internal gas pressure. This stability window narrows with increasing temperature and cluster size and converges to a characteristic range of 2–3, which is consistent with large-scale MD simulation observations. Furthermore, we extended the critical mutation criterion to finite-temperature regimes by establishing the dependence of the He/V threshold on temperature and size, thereby enabling the predictive estimation of mutation behavior across a broad thermodynamic landscape.

Collectively, these insights deepen our understanding of helium-induced defect evolution in tungsten and offer key input parameters for mesoscale and continuum models. The quantitative criteria established here will pave the way for more accurate predictions of helium bubble evolution, trap mutation onset, and defect-driven degradation in plasma-facing materials for fusion reactors. Although this study focuses on tungsten, the framework established here can be extended to other bcc metals and plasma-facing materials such as molybdenum and tantalum. For alloyed or defect-rich systems, it would be necessary to use accurate interatomic potentials for multi-element interactions and explicitly model microstructural features such as solute atoms, grain boundaries, and dislocations. These adaptations will further enhance the predictive capability of the framework in complex material environments.

## Data Availability

The raw data supporting the conclusions of this article will be made available by the authors upon request.

## Abbreviations

The following abbreviations are used in this manuscript:

MD	Molecular dynamics
MS	Molecular statics
Vac	Vacancy
SIA	Self-interstitial atom
He	Helium
He/V	helium-to-vacancy ratio
